# The Hog1 MAP Kinase Promotes the Recovery from Cell Cycle Arrest Induced by Hydrogen Peroxide in *Candida albicans*

**DOI:** 10.3389/fmicb.2016.02133

**Published:** 2017-01-06

**Authors:** Inês Correia, Rebeca Alonso-Monge, Jesús Pla

**Affiliations:** Departamento de Microbiología II, Facultad de Farmacia, Universidad Complutense de MadridMadrid, Spain

**Keywords:** *Candida albicans*, cell cycle, oxidative stress, signaling, MAPK

## Abstract

Eukaryotic cell cycle progression in response to environmental conditions is controlled via specific checkpoints. Signal transduction pathways mediated by MAPKs play a crucial role in sensing stress. For example, the canonical MAPKs Mkc1 (of the cell wall integrity pathway), and Hog1 (of the HOG pathway), are activated upon oxidative stress. In this work, we have analyzed the effect of oxidative stress induced by hydrogen peroxide on cell cycle progression in *Candida albicans*. Hydrogen peroxide was shown to induce a transient arrest at the G1 phase of the cell cycle. Specifically, a G1 arrest was observed, although phosphorylation of Mkc1 and Hog1 MAPKs can take place at all stages of the cell cycle. Interestingly, *hog1* (but not *mkc1*) mutants required a longer time compared to wild type cells to resume growth after hydrogen peroxide challenge. Using GFP-labeled cells and mixed cultures of wild type and *hog1* cells we were able to show that *hog1* mutants progress faster through the cell cycle under standard growth conditions in the absence of stress (YPD at 37°C). Consequently, *hog1* mutants exhibited a smaller cell size. The altered cell cycle progression correlates with altered expression of the G1 cyclins Cln3 and Pcl2 in *hog1* cells compared to the wild type strain. In addition, Hgc1 (a hypha-specific G1 cyclin) as well as Cln3 displayed a different kinetics of expression in the presence of hydrogen peroxide in *hog1* mutants. Collectively, these results indicate that Hog1 regulates the expression of G1 cyclins not only in response to oxidative stress, but also under standard growth conditions. Hydrogen peroxide treated cells did not show fluctuations in the mRNA levels for *SOL1*, which are observed in untreated cells during cell cycle progression. In addition, treatment with hydrogen peroxide prevented degradation of Sol1, an effect which was enhanced in *hog1* mutants. Therefore, in *C. albicans*, the MAPK Hog1 mediates cell cycle progression in response to oxidative stress, and further participates in the cell size checkpoint during vegetative growth.

## Introduction

*Candida albicans* is a pathogenic yeast of great clinical significance (Brown et al., 2012). This fungus colonizes mucosal surfaces of humans, where it behaves as a harmless commensal, but is able to cause a range of diseases under situations that compromise host defenses. Candidiasis, as these diseases are collectively called, can be life-threatening among individuals with an impaired immune system (Pfaller and Diekema, 2007). A biological characteristic of *C. albicans* is its ability to develop different morphologies (yeast, hypha, pseudohypha, and chlamydospore), and engage in morphogenetic transitions (i.e., white-opaque) under certain environmental conditions. This trait contributes to its versatility as a pathogen (Sudbery et al., 2004; Whiteway and Bachewich, 2007; Berman, 2012; Sellam and Whiteway, 2016). Morphology influences virulence, as hyphal-defective mutants are frequently less virulent in animal models of infection (Lo et al., 1997; Alonso-Monge et al., 1999; Saville et al., 2006).

Although it is an essential biological process, the cell cycle has received relatively little attention in *C. albicans* compared to other fungal models (Berman, 2006; Correia et al., 2010). For studies of the eukaryotic cell cycle, the yeast *Saccharomyces cerevisiae* is frequently used as a model organism (Berman and Sudbery, 2002). The cell cycle culminates in mitosis and cytokinesis and comprises two gap periods before the DNA synthesis period (called the S phase): the G1 phase that precedes S phase, and the G2 phase that follows S phase. A G0 (or latency) phase of variable length can be also observed (Gray et al., 2004). Given the crucial role of the cell cycle for any living cell, distinct checkpoints ensure that all cellular events take place sequentially after certain requirements have been met, or otherwise a temporal arrest occurs. A checkpoint, named *START*, is present at the end of the G1 phase to ensure that an adequate cell size is achieved. This checkpoint is coordinated with environmental conditions such as nutrients, salts and temperature (Rupes, 2002). Another checkpoint is present before entry into mitosis, where cell size and correct DNA duplication are controlled. Recent studies indicate that organization of the septin proteins, and the curvature formed in the cell membrane are crucial to control this checkpoint (Kang and Lew, 2016). In *S. cerevisiae*, an additional morphogenetic checkpoint exists that blocks division in un-budded cells (Lew and Reed, 1995). Progression through the cell cycle is governed by the action of cyclins, whose abundance is cell cycle-dependent and determined by their synthesis and degradation rates. Cyclins interact with a single cyclin-dependent kinase (CDK) (named Cdc28 in *S. cerevisiae*), and can be classified in G1 cyclins (Cln1, Cln2 and Cln3), S-phase cyclins (Clb5 and Clb6), and mitotic cyclins (Clb1-4) (Bloom and Cross, 2007). In *C. albicans*, the G1 cyclins homologous to those of *S. cerevisiae* are Ccn1, Cln3, and Hgc1, and they appear to have a specific role in the control of morphogenesis. Ccn1 is important for the maintenance of hyphal growth (Loeb et al., 1999), Hgc1 is a hypha specific G1 cyclin (Zheng et al., 2004), and *CLN3* is an essential gene that regulates cell size (Chapa y Lazo et al., 2005). *Candida albicans* has only two B-type cyclins, Clb2 and Clb4 (the first one being essential for growth), which negatively regulate polarized growth (Bensen et al., 2005).

Cell cycle progression is regulated by environmental signals (Waltermann and Klipp, 2010). MAPK pathways are key elements of this control given their role in sensing and responding to external stimuli (Chen and Thorner, 2007). In *S. cerevisiae*, three pathways have been shown to mediate cell cycle progression: the pheromone response pathway, the cell integrity pathway and the HOG pathway. The pheromone response pathway is responsible for arresting cells prior to mating in response to pheromones through the action of Fus3 (the MAP kinase which is the homolog of Cek2 in *C. albicans*) and Kss1 (the MAPK kinase which is the homolog of Cek1 in *C. albicans*) (Elion, 2000). The *PKC1-*mediated cell integrity pathway controls the activation of the Slt2 MAP kinase (homolog of *C. albicans* Mkc1), and this occurs in a cell cycle-dependent fashion (Marini et al., 1996; Zarzov et al., 1996). Activation of the PKC pathway leads to the expression of cell wall enzymes through the action of the transcription factors Rlm1 and the Swi4/Swi6 cell cycle box (SCB)-binding factor (SBF) (Madden et al., 1997; Jung and Levin, 1999; Baetz et al., 2001). The SBF complex is the main activator of a set of genes involved in the G1/S-phase transition or START (Breeden, 1996).

Since its discovery more than 20 years ago (Brewster and Gustin, 2014), the HOG pathway has been extensively studied in *S. cerevisiae*. This pathway is largely responsible for growth under high osmolarity, a situation of great significance for environmental yeasts (Hohmann, 2002). Under these conditions, the HOG pathway controls progression through the S phase (Yaakov et al., 2009) and also regulates exit from mitosis (MEN) (Reiser et al., 2006; Radmaneshfar et al., 2013). In *S. cerevisiae*, osmotic stress induces a transient cellular arrest. Hog1, the canonical MAPK of the HOG pathway, has been shown to be involved in G1 progression (Belli et al., 2001). This conclusion is in agreement with a prolonged G1 arrest observed in *hog1* mutants after osmotic challenge (Migdal et al., 2008). Hog1 phosphorylates the CDK inhibitor Sic1, reducing the expression of the *CLN1* and *CLN2* cyclins and, consequently preventing cell cycle progression upon osmotic challenge (Escoté et al., 2004). Hog1 has also an important role in G2 phase through stabilization of Swe1, a protein kinase which needs to be degraded for cell cycle progression to occur (Clotet et al., 2006). In addition to osmotic stress, exposure to arsenite also leads to a Hog1-dependent G1 and G2 delay (Migdal et al., 2008).

There are only a few studies on cell cycle regulation by MAP kinases in *C. albicans* (Côte et al., 2009, 2011). This is despite the fact that the HOG pathway is a major determinant of pathogenicity in this fungus and is involved in morphogenesis, cell wall biogenesis, stress response and virulence (Alonso-Monge et al., 1999; Arana et al., 2007; Román et al., 2007). Here we have undertaken the analysis of the role of the HOG pathway in the control of the cell cycle in *C. albicans*, and in particular, in the response to oxidative stress induced by hydrogen peroxide. We show that *hog1* mutants are defective in the recovery from a hydrogen peroxide-mediated cell cycle arrest, implicating that the HOG pathway controls cell cycle progression under stress conditions in *C. albicans*.

## Materials and methods

### Strains and growth conditions

Yeast strains used in this study are listed in Table 1. *C. albicans* was routinely grown at 37°C in YPD medium (1% yeast extract, 2% peptone, 2% glucose) and kept at 4°C for short-term storage. Culture growth was assessed through the measure of absorbance at 600 nm. Cell viability was evaluated by counting colony forming units (CFUs). Cells treated (or not) with H_2_O_2_ (1 mM) were plated on YPD solid medium in serial dilutions. Plates were then incubated at 37°C for at least 48 h, and then colonies were counted.

**Table 1:** **Strains of microorganisms used in this study**.

**Strain**	**Genotype and strain background**	**Abbreviated nomenclature in text and figures**	**Source**
SC5314	Clinical isolate	SC5314 (wild type)	Gillum et al., 1984
CAF2	*ura3::imm434/ura3::imm434-URA3 IRO1/iro1::imm434*	CAF2 (wild type)	Fonzi and Irwin, 1993
CAI4	*ura3::imm434/ura3::imm434 iro1::imm434/iro1::imm434*	CAI4 (wild type)	Fonzi and Irwin, 1993
CK43B-16	[CAI4] *cek1::hisG-URA3-hisG/cek1::hisG*	*cek1*	Csank et al., 1998
CM1613	[CAI4] *mkc1::hisG-URA3-hisG/mkc1::hisG*	*mkc1*	Navarro-García, 1995
HI3	[CAI4] *hog1::hisG/hog1::hisG-URA3-hisG*	*hog1*	Prieto et al., 2014
HI7	[CAI4] *hog1::hisG/hog1::hisG*		
COA6	[CAF2] *ADH1/adh1::tTA pTet-*GFP*-SAT1*	CAF2-GFP	Prieto et al., 2014
SC2H3	SN152 *(arg4Δ/arg4Δ leu2Δ/leu2Δ his1Δ/his1Δ URA3/ura3Δ::imm434 IRO1/iro1::imm434) 5xLexAOp-ADH1b/HIS1 5xLexAOp-ADH1b/lacZ*	SC2H3	Stynen et al., 2010
CCS1	[CAI4] *ADH1/adh1::tTA pTet-SOL1-myc-URA3*	CAI4 pNRU-*SOL1*	This study
CCH46	[*hog1 ura3*] *ADH1/adh1::tTA pTet-SOL1-myc-URA3*	*hog1* pNRU-*SOL1*	This study

### Molecular biology procedures and plasmids construction

The integration of genetic constructs in *C. albicans* was achieved by the transformation system developed by Köhler et al. (1997). To achieve *SOL1* ectopic expression and myc fusion, the *SOL1* ORF was introduced in the pNRU-RFP plasmid (Correia et al., 2016) after digestion with the *Sal*I-*Not*I restriction enzymes. *SOL1* was amplified by PCR from DNA of the clinical isolate SC5314, using the primers o-Sol1myc-up (GATGTCGACAATGTCCTCTTCTAATGATACACCATC) and o-Sol1myc-lw (TTCGCGGCCGCTTCTCGAGGGTATATTATCAAACGATAATCTCTTTGG). PCR products were sub-cloned in the intermediate pGEM-T plasmid (Promega) and sequenced.

For the analysis of a putative interaction between Sol1 and Hog1, a *C. albicans* two-hybrid system was used as described previously (Stynen et al., 2010; Correia et al., 2016). At least 20 clones from each transformation were analyzed for growth on histidine and methionine depleted medium at 37°C for up to 5 days, and no differences between clones were observed (Supplementary Figure 2). Representative clones from each strain were selected for comparative growth. Primers used for construction of bait and prey plasmids are listed in Table 2.

**Table 2:** **Primers used for the construction of strains for two-hybrid analysis**.

**Primer**	**Sequence 5′-3′**
o-HOG1-Prey-up	CAAAGGCCTATGTCTGCAGATGGAGAATTTACAAG
o-HOG1-Prey-lw	CTTGGCGCGCCTTAAGCTCCGTTGGCGG
o-SOL1-Bait-up	CAAGCTAGCATGTCCTCTTCTAATGATACACCATC
o-SOL1-Bait-lw	CTTAGGCCTTTATATATTATCAAACGATAATCTCTTTGG

### Protein extracts and immunoblot analysis

The yeast samples for protein immune detection were collected on ice and processed for protein extraction as previously described (Martín et al., 2000). The protein concentration of the supernatants was measured at 280 nm and normalized with loading buffer. Membranes were probed with anti-phospho-p38 and anti-phospho-p44/42 (Cell Signalling Technology, Inc.), for detection of P-Hog1 and P-Mkc1/P-Cek1 respectively. Hog1 protein was detected by the anti-ScHog1(y-215) polyclonal antibody (Santa Cruz Biotechnology). Mkc1 and Cek1 protein levels were determined using previously described polyclonal sera (Navarro-García et al., 2005; Román et al., 2005). For myc detection, an anti-myc clone 4A6 (Millipore) was used. Western blots were developed according to the manufacturer's instructions using the Hybond ECL kit (Amersham Pharmacia Biotech) or the Quantitative Fluorescent Imaging System Odyssey from LI-COR.

### Synchronization method for cell cycle analysis

Cell cycle synchronization was achieved by elutriation using an Avanti® J Series instrument from Beckman Coulter with a JE-5.0 elutriator rotor. *C. albicans* cells were grown for 6.5 h at 37°C and refreshed in two plastic flasks with 1L of pre-warmed YPD each, at O.D. = 0.2. The cultures were allowed to grow at 37°C until O.D. = 1 was reached. The 2 L of exponentially growing cells were then introduced into the elutriation chamber at 4°C, and cells were recovered, on ice, by increasing the flow rate. The low temperature was used to arrest cell division during the elutriation process. The efficiency of elutriation was confirmed firstly through microscopic analysis (<10% budded cells) and secondly by measuring the DNA content by flow cytometry upon fixation and staining of the cells with propidium iodide (PI). For simultaneous synchronization each strain was grown separately at 37°C to O.D. = 1 and mixed in equal proportions just before elutriation. The synchronized culture (composed of a single strain or of two strains) was finally divided into two pre-warmed flasks for differential treatment, and incubated at 37°C. Samples were taken at different time points upon treatment, as well as before and after elutriation as a technical control.

### Flow cytometry analysis

Samples were fixed with 70% ethanol (5 min at RT), treated with RNase (30 min at 37°C) and stained with PI at a final concentration of 0.0005%. For simultaneous cell cycle analysis where the wild-type CAF2 was tagged with the green fluorescent protein (CAF2-GFP), the fixation protocol was optimized to avoid loss of fluorescence intensity: cells were first incubated with formaldehyde at 4% for 1 h on ice, followed by 1 min contact with 70% ethanol. Cells were washed with PBS at each step of the fixation treatment and pipette and vortex were used for re-suspending cells after ethanol fixation in order to avoid possible clumps. DNA quantification (measured by PI intensity) was assessed by flow cytometry with a FACScan (Becton Dickinson) from the UCM (Universidad Complutense de Madrid) cytometry service, and profiles were analyzed with the Flowing Software 2.5.1. 10.000 events were analyzed by flow cytometry.

### Microscopy

Microscopic observation was made from fresh cell preparations by phase contrast with an Olympus BH-2 microscope at a magnification of 40x (unless otherwise stated). Pictures were obtained by a Panasonic CCD camera coupled to the microscope using DScaler software, or by a digital camera Panasonic Lumix DMC-G1K lens kit. Fluorescence microscopy images from cells treated for flow cytometry analysis were obtained from a Nikon Eclipse TE2000-U coupled with a Hamamatsu ORCA-ER CCD camera. The filters used were Nikon B-2E/C for green (GFP) and Nikon G-2A for red (PI). All pictures from each experiment were taken (gain and exposure) and processed (brightness and contrast) equally, using the Aquacosmos 1.3 program and Adobe Photoshop CS5.

### Quantitative reverse transcription-PCR assay

RNA was extracted from synchronized cultures of strains CAF2 and *hog1* growing at 37°C in YPD medium in the presence or absence of 1 mM H_2_O_2_. Total RNA was isolated from cells by the “mechanical disruption protocol” using the RNeasy MINI kit (Qiagen, Hilden, Germany), following the instructions provided by the manufacturer. RNA concentrations were determined by measuring absorbance at 260 nm. First-strand cDNAs were synthesized from 2 μg of total RNA, using the Reverse Transcription System (Promega) and following the recommendations of the manufacturer. As controls for genomic contamination, the same reactions were performed, but in the absence of reverse transcriptase. Q-RT-PCR assay was performed by following a previously described protocol (Garcia et al., 2004) using the SYBR Green Universal Master Mix (Applied Biosystems). The conditions for Real-time PCR were selected according to the Universal conditions (default conditions) recommended by the manufacturer. Each cDNA from two independent experiments was assayed in triplicate PCR reactions. Basic analysis was performed using the SDS 1.9.1 software (Applied Biosystems). For quantification, the abundance of each gene was normalized to the standard transcript of *ACT1* (i.e., actin transcript level served as a reference). The primers used are listed in Table 3.

**Table 3:** **Primer sequences for RT-PCR experiment**.

**Primer**	**Sequence 5′-3′**
o-ACTQTup	TGGTGGTTCTATCTTGGCTTCA
o-ACTQTlw	ATCCACATTTGTTGGAAAGTAGA
o-CLN3-QTup	CCTGTATCCAATAGCACTAGCCCT
o-CLN3-QTlw	CTCATTTTCCGAAAATATCTGATCAG
o-PCL2-QTup	TGATGCAAAAATCTAAAGCCGTT
o-PCL2-QTlw	TGTAAGGATAATTTCGATTCGATCG
o-HGC1-QTup	CATCTTCTAATGCAAATACACCAAGTTC
o-HGC1-QTlw	AGGTGTCATACCTAATGGAGTTGTTG
o-SOL1up-PCRQ	TGGTAGATGTCATAACTCTTTCACG
o-SOL1lw-PCRQ	AACTCATTAACCTTCTTCAAATCAAAT

### Bioinformatics and statistical analysis

Results were calculated and presented as the mean of the measured values ± the standard error of the mean (SEM). The SPSS software was used for the statistical analysis of the data. Significant differences were detected using 2-way ANOVA and Sidak's multiple comparisons test. The significance is represented in the figures as ^*^ when *p* < 0.05 and ^**^ for *p* < 0.001.

## Results

### Hydrogen peroxide arrests the cell cycle in the G1 phase

In *S. cerevisiae*, mutants defective in the superoxide dismutase gene (*SOD1*) display a delay in G1 (Lee et al., 1996). Depletion of gluthatione by addition of diethylmaleate (DEM) to exponentially growing cells also results in a G1-to-S delay (Wanke et al., 1999). Therefore, oxidative stress plays an important role in the regulation of cell cycle in this yeast. As oxidative stress is an essential environmental signal for human pathogens such as *C. albicans*, we wondered whether oxidative stress could interfere with the cell cycle in this fungus.

**Figure 1:**
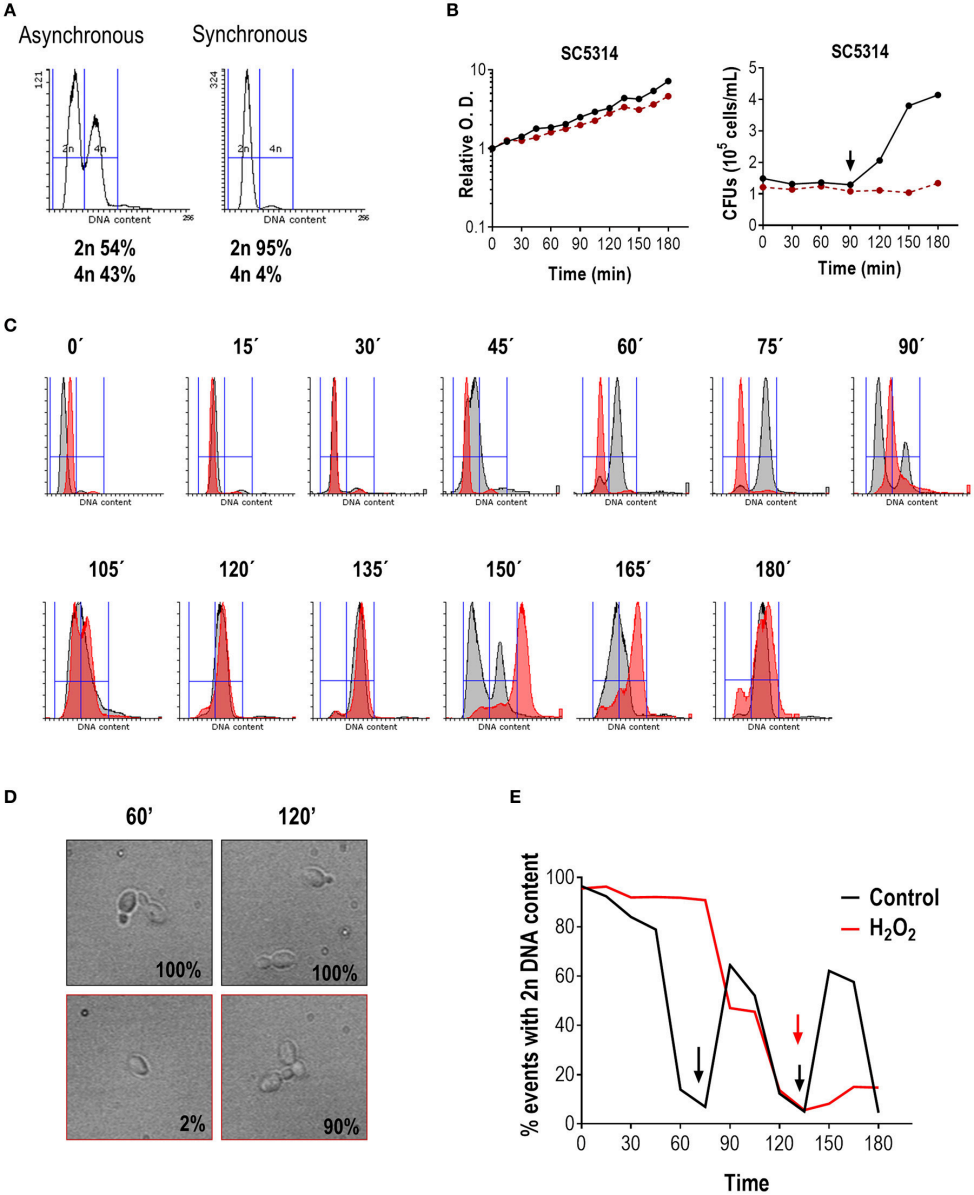
**Hydrogen peroxide arrests ***C. albicans*** in G1. (A)** Representative flow cytometry analysis of SC5314 before and after elutriation. Histograms represent the number of events (cells) in the y-axis vs. 2n or 4n DNA content (x-axis). The percentage of cells from each population with 2n or 4n DNA content are indicated. **(B)** Synchronous cultures of SC5314 were released in YPD (black line) or YPD plus 1 mM H_2_O_2_ (red line) and relative O.D. was plotted against time (in min) (left panel) or CFUs vs. time (in min) (right panel). The arrow indicates CFUs doubling, which correlates with cytokinesis. **(C)** Cultures released in YPD (gray) or YPD plus 1 mM H_2_O_2_ (red) were followed over time. Flow cytometry histograms with the corresponding time point are shown. **(D)** In parallel, budding percentage was assessed by counting using an optical microscope. Representative pictures of YPD without (upper row, black frame) and with 1 mM H_2_O_2_ (lower row, red frame) are shown, and budding percentage indicated at 60 and 120 min. **(E)** Graph indicating the percentage of cells with 2n DNA content vs. time (in min) of released cells in YPD (black line) and in the presence of H_2_O_2_ (red line). Arrows indicate 4n DNA content.

For this purpose, wild type cells (strain SC5314) exponentially growing at 37°C to O.D. = 1 were synchronized by elutriation and the DNA content analyzed by flow cytometry. As shown in Figure 1A, the asynchronous culture (before elutriation) displayed a characteristic histogram, whereby two peaks of DNA content (2n for G1 cells and 4n for G2 or M cells) were easily discernible. Synchronization via elutriation allowed recovery of more than 95% of un-budded G1 (or G0) cells with 2n DNA content. The synchronized G1 culture was then divided into two flasks and one of them was treated with 1 mM H_2_O_2_. This concentration of hydrogen peroxide was determined as being optimal to induce an effect on cell cycle progression and MAPKs phosphorylation (Supplementary Figure 1), but without significantly altering cell viability. Cells were allowed to grow at 37°C and samples were taken every 15 min for 3 h. Progression of both cultures was followed by O.D. measurements and CFUs counts (Figure 1B). The O.D. increased steadily in the next 180 min for both cultures, although the increase was slightly lower for hydrogen peroxide treated cells. CFU remained stable until 90 min and doubled after in untreated cultures correlating with cytokinesis. No cytokinesis was detected in the hydrogen peroxide challenged culture. After release from elutriation at 37°C, DNA duplication began in untreated controls at 45 min (Figure 1C) and 15 min later (at 60 min) almost all cells were already displaying a 4n DNA content, correlating with 100% of cells showing emergence of a bud (Figure 1D). In contrast, in hydrogen peroxide treated cells DNA started to duplicate around 90/105 min upon synchronization. At 75 min, 87% of non-treated cells displayed a 4n DNA content, while in treated cells it took 120 min to reach an equivalent percentage of cells with 4n DNA content (84%), and at that time almost 100% cells in treated cultures exhibited small buds. Therefore, the addition of H_2_O_2_ to the SC5314 strain led to a delay of almost 1 h in cell cycle progression. While un-treated cells were able to return to the G1 phase at 90 min and 150 min (Figure 1E), only 15% of the oxidative challenged cells presented with a 2n DNA content at the end of the experiment (180 min). We conclude from this assay that hydrogen peroxide arrests SC5314 *C. albicans* cells specifically at the G1 phase of the cell cycle.

### Hog1 is involved in resumption of growth after cell cycle arrest

**Figure 2:**
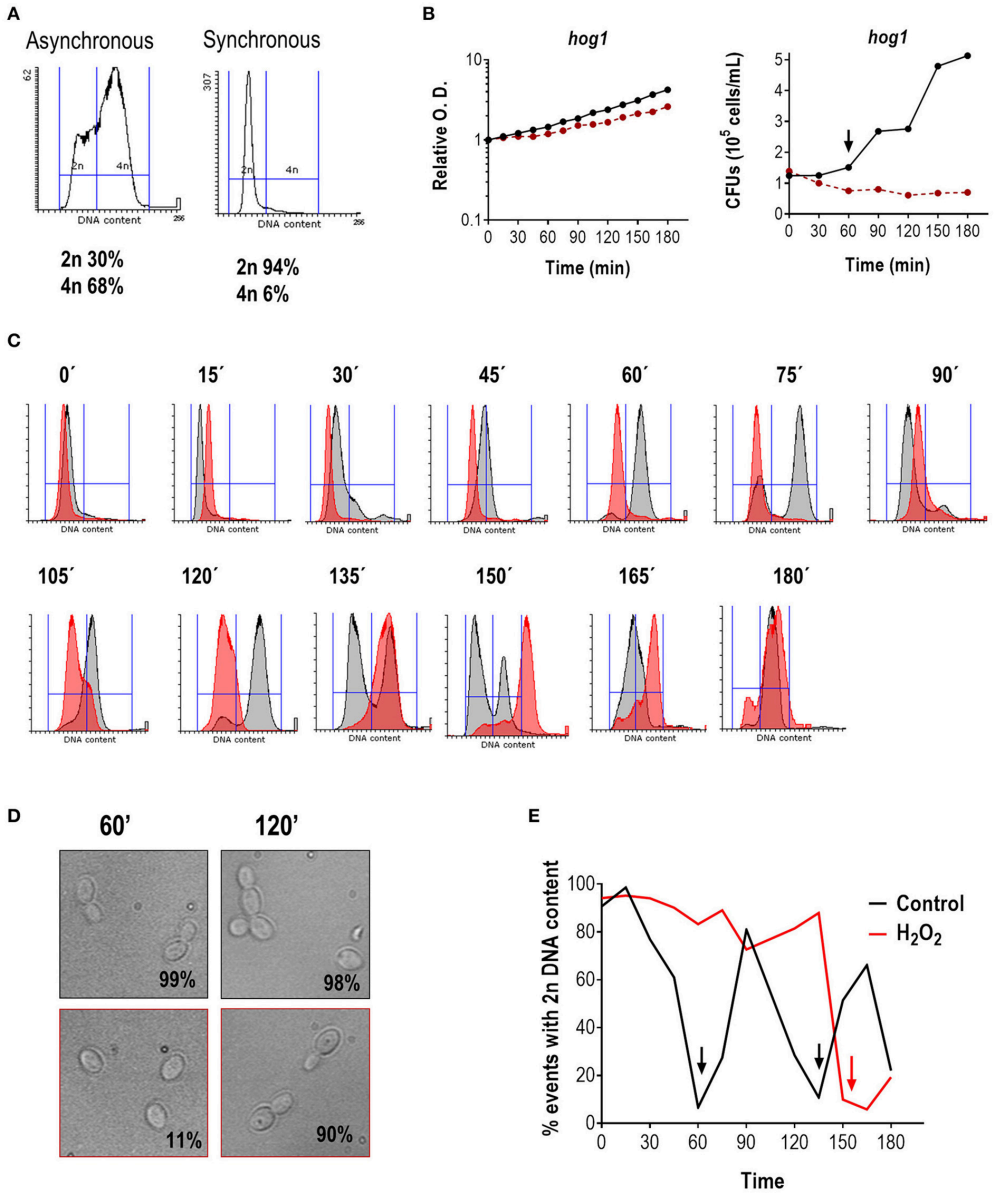
**Effect of H_**2**_O_**2**_ on cell cycle progression in the ***hog1*** mutant. (A)** Flow cytometry analysis of *hog1* mutant cells before and after elutriation. The percentage of cells with 2n and 4n DNA content are indicated. **(B)** After elutriation, G1 synchronized cells were released in YPD at 37°C with or without 1 mM H_2_O_2_. The growth was followed over time and depicted as relative O.D. (left panel) or CFUs vs. time (right panel). The black line represents cultures without stress, while the red line represents cultures in the presence of H_2_O_2_. The arrow marks cytokinesis. **(C)** Histograms of cultures released in the presence (red) or absence (gray) of oxidative stress at the indicated time. **(D)** Representative pictures of the culture without stress (upper row, black frame) or with an oxidative agent (1 mM H_2_O_2_, lower row, red frame). The percentage of budding is indicated for each condition. **(E)** The percentage of cells with 2n DNA content is plotted vs. time for cultures released with and without stress. Arrows indicate 4n DNA content.

In *S. cerevisiae, HOG1* mediates cell cycle arrest in response to osmotic stress (Escoté et al., 2004). We therefore asked whether Hog1 could accomplish a similar function in *C. albicans* in response to oxidative stress. We performed a similar experiment to the one shown in Figure 1. As shown in Figure 2, the *hog1* mutant strain follows a normal cell cycle progression in the absence of oxidant. The mutant was able to enter the S/G2 phase and returns to the G1 phase twice (at 90 and 150 min), which is equivalent to what occurs for the wild type strain (Figure 2E). However, DNA duplication in *hog1* cells seemed to begin slightly earlier than in the wild type: at 30 min 20.1% of the *hog1* cells had 4n of DNA content (vs. 9.6% in the wild type Figure 1C). The percentage of *hog1*cells with 4n DNA content reached 89.9% at 60 min (Figure 2C; at the same time point 77.9% of wild type cells had 4n DNA content). Furthermore, more cells displayed bud emergence at 30 min in the mutant (28% budded cells in *hog1* cells vs. 2% in the wild type) (data not shown), and bigger buds at 60 min compare to wild type buds (compare Figures 1D, 2D). When synchronized cells of the *hog1* mutant were grown in the presence of peroxide, cells arrested the cell cycle and were able to resume growth afterwards. However, the exit from G1 was substantially delayed in the *hog1* mutant compared to the wild type (compare Figures 1C, 2C): while at 120 min 84% of wild type cells were already in G2 (4n DNA content), at the same time point 82% of *hog1* were still in G1 (2n DNA content), and only at 150 min the majority of *hog1* mutant cells (89%) exhibited 4n DNA content. At the end of the experiment (180 min) 19% of the *hog1* cells were able to return to the G1 phase. Analysis of O.D. and cell viability (CFUs count) during cell cycle progression revealed that the final O.D.s for cells (wild type and mutant) treated with H_2_O_2_ were lower (roughly half) than without the oxidant (Figures 1B, 2B). Interestingly, CFUs from un-treated *hog1* mutant cultures increased earlier than CFUs from un-treated wild type cultures (60 vs. 90 min), consistent with the cytometry profile and with the notion that the *hog1* mutant seems to progress more rapidly through the cell cycle.

In order to confirm that the *hog1* mutant displays a delay in resumption of growth upon an oxidative stress-dependent cell cycle arrest, we performed simultaneous analysis of the *hog1* mutant and wild type population. For this purpose, we integrated a genetic construction carrying the green fluorescent protein (GFP) at the *ADH1 locus* of the CAF2 strain, which allowed differential microscopy observation and flow cytometry analysis of mixed cultures (wild type cells were labeled by GFP, while mutant cells were not). For simultaneous synchronization the strains CAF2-GFP and *hog1* were grown separately at 37°C until O.D. = 1 and then mixed in equal proportions before elutriation. The obtained synchronous cells were then divided in two flasks and treated (or not) with hydrogen peroxide as described above. Samples were taken before and after elutriation, and in 15 min intervals upon stress for fluorescence microscopy and FACS analysis.

**Figure 3:**
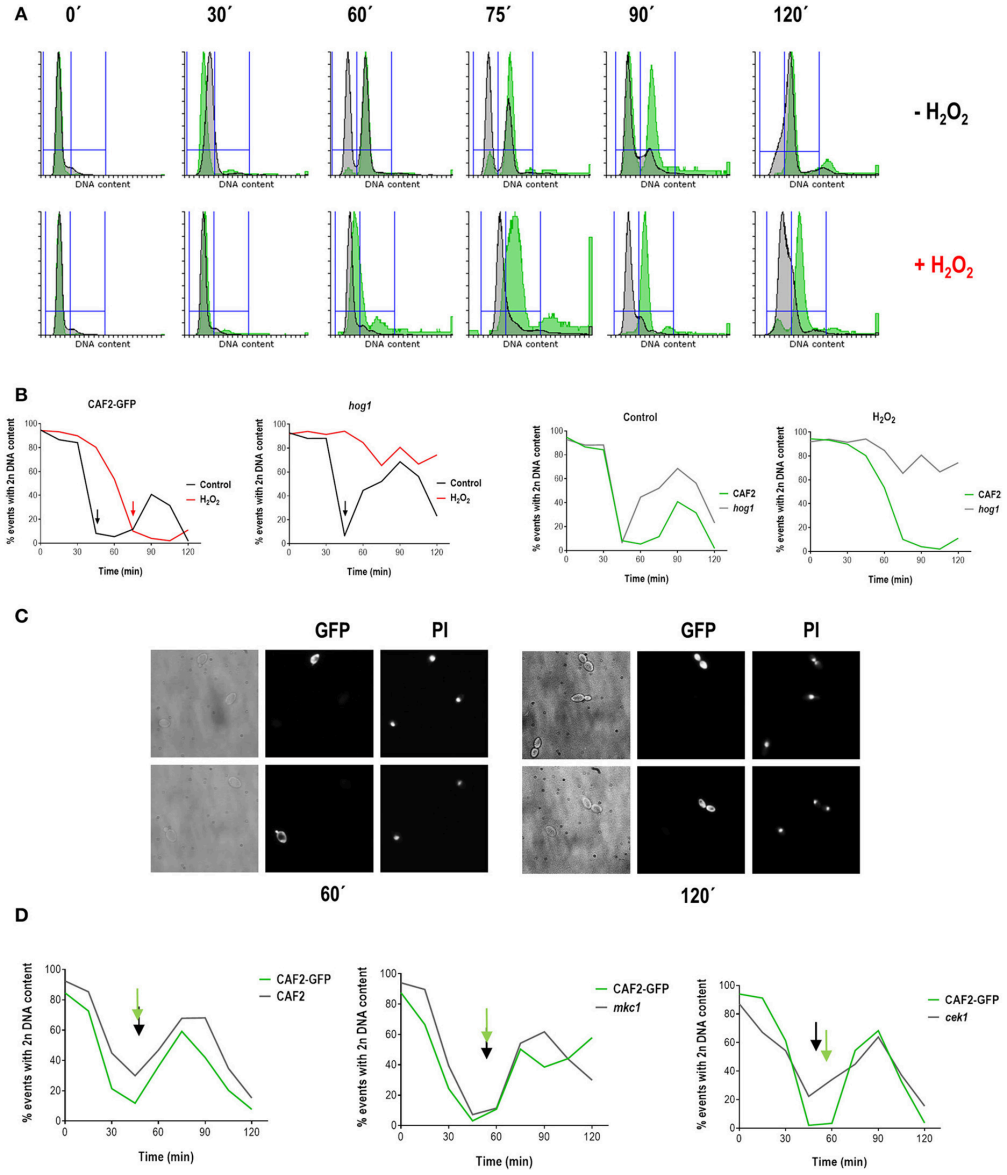
**Analyses of cell cycle progression in mixed (wild type/mutant) ***C. albicans*** cultures**. CAF2-GFP and *hog1* strains were grown separately until exponential phase and cultures from the two strains (comprising similar number of cells) were mixed before elutriation. Elutriated recovered cells corresponding to G1 phase were grown at 37°C in the presence or absence of 1 mM H_2_O_2_. Samples were collected at different time points for FACS analysis. **(A)** Histograms from mixed cultures after elutriation are presented. DNA content of the CAF2-GFP strain is shown in green, while DNA content from the *hog1* mutant is shown in gray. The upper row shows histograms from non-treated cells, while the lower row shows histograms for H_2_O_2_ treated cells. **(B)** Graphs represent the percentage of cells with 2n DNA content over time for the indicated strains and conditions (control means YPD without stress and H_2_O_2_ means YPD supplemented with 1 mM H_2_O_2_). Arrows indicate a 4n DNA content. **(C)** Samples were collected and cells visualized with optical and fluorescence microscopy. The pictures show cells from a CAF2-GFP/*hog1* co-culture treated with 1 mM H_2_O_2_ for 60 and 120 min, and stained with propidium iodide (PI) for visualization of nuclei. **(D)** Similar experiments were performed with the indicated mixed cultures and percentage of cells with 2n DNA content is shown vs. time. Arrows signpost valley in 2n DNA content.

The simultaneous synchronization of both strains allowed us to confirm some of the previously suggested results. Firstly, *C. albicans* cell cycle progression is arrested by oxidative stress (Figures 3A,B): at 60 min 86% of non-treated cells were already in G2, while only 29% of treated cells presented with a 4n DNA content. This arrest is independent of Hog1 as *hog1* cells are able to arrest cell cycle progression at G1: at 60 min, 53% of non-treated cells were with 4n DNA content and already returning to G1 (see *hog1* graphic in Figure 3B), while only 13% of treated cells presented with a 4n DNA content. Secondly, *hog1* cells recover later from oxidative stress-mediated cell cycle arrest compared to wild type cells (Figures 3A,B). This is better observed at 90 min, where 84% of wild type cells recovered upon treatment had already doubled their DNA content, while 81% of treated *hog1* cells remained in G1.The delay in cell cycle progression was, in parallel, observed with fluorescence microscopy (Figure 3C). At 60 min the green-fluorescent cells (CAF2-GFP tagged cells) presented a small bud correlating with 4n DNA content displayed in the histogram (Figure 3A). The non-green fluorescent cells (that is the *hog1* mutant) remained as unbudded cells at the same time point. Likewise, at 120 min some cells presented fully developed daughter cells with their individual nucleus (these cells overlap with green-fluorescent cells or CAF2-GFP), while non-green-fluorescent *hog1* cells exhibited a single nucleus despite bud emergence. The *hog1* cells were unable to bypass the G1/S checkpoint before the end of the experiment (only 23% of the *hog1* cells displayed a 4n DNA content at 120 min). We conclude from these experiments that *hog1* cells display a defect in resuming growth after a cell cycle arrest by hydrogen peroxide. Finally, under non-stressful conditions, cell cycle transitions seem to occur more rapidly in the *hog1* mutant compared with the wild type strain. Although in this assay we were not able to identify a faster entry into G2 by the *hog1* mutant (because the experiment lasted 120 min and not 180 min). While at 60 min 45% of *hog1* cells had already entered into the G1 phase (see *hog1* graphic in Figures 3A,B), 86% of CAF2 cells were still with 4n DNA content. This behavior was specific to *hog1* mutants. By using the same methodological approach (mixed population between labeled CAF2-GFP cells and unlabeled MAPK mutants) we were able to analyze cell cycle progression under standard growth conditions of *mkc1* and *cek1* mutant cells, and no differences were found in cell cycle progression compared to the wild typ. Altogether, our data support a role for Hog1 in cell cycle progression under normal growth conditions, and in cell cycle re-start after oxidative stress-induced arrest.

### Hydrogen peroxide-mediated MAPK activation occurs at all stages of the cell cycle

**Figure 4:**
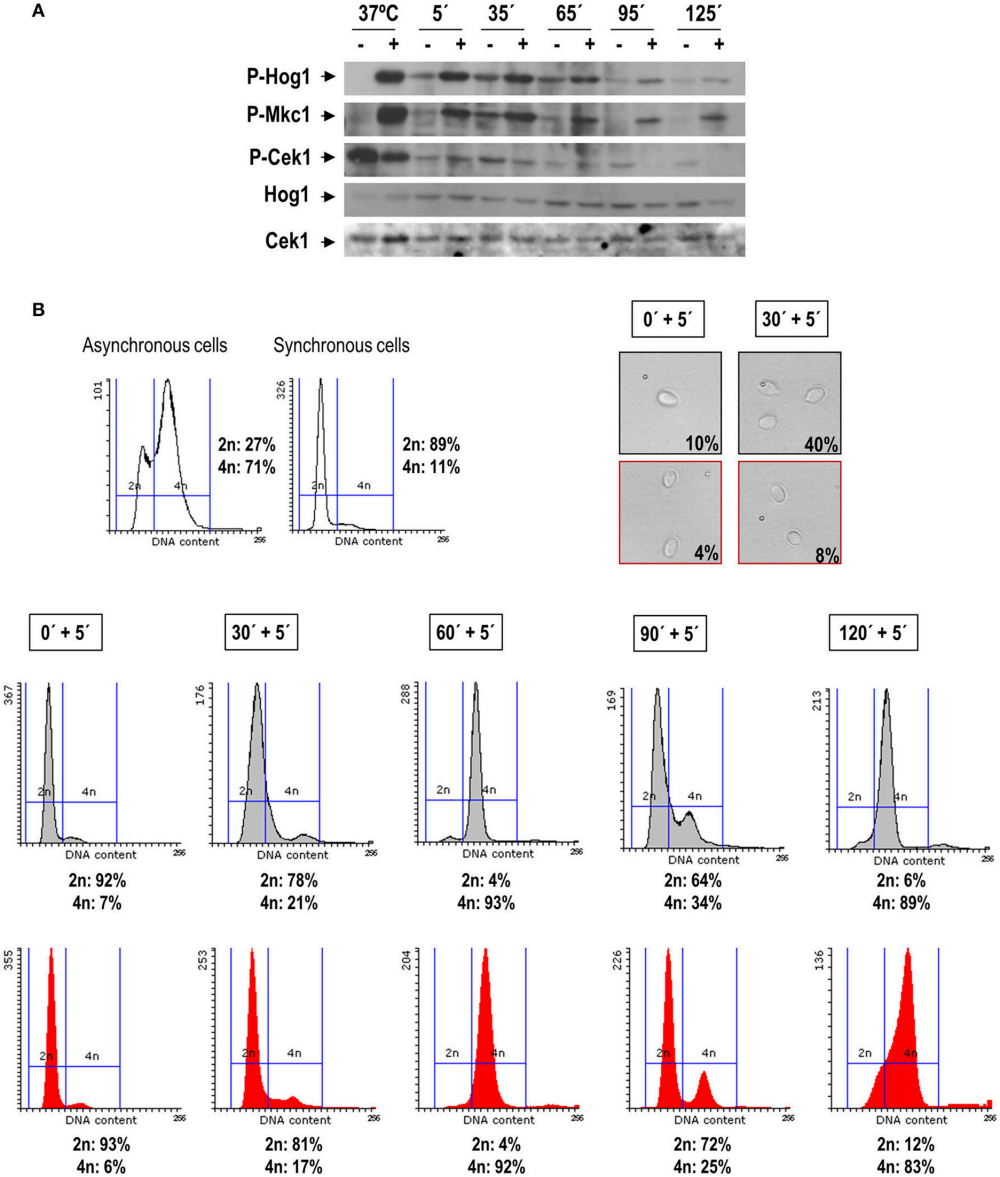
**H_**2**_O_**2**_ triggers MAPK phosphorylation along the cell cycle**. Exponentially growing cells from the SC5314 strain were synchronized through elutriation. G1 synchronous cells were recovered and let to resume growth in YPD at 37°C. Samples were taken at different time points, separated in two flasks and treated or not with 5 mM H_2_O_2_ for 5 min. Cells were recovered and processed for detection of MAPK phosphorylation **(A)** and for FACS and microscopy analysis **(B)**. Budding percentage was assessed by visual count and indicated for each time points. Phosphorylation of Hog1 (indicated as P-Hog1) was detected using anti-phospho-p38 antibody. Mkc1 and Cek1 phosphorylation (P-Mkc1 and P-Cek1) was detected by anti-phospho-p42/44 antibody. Non-phosphorylated forms of the proteins were detected using specific antibodies. The sample 37°C refers to an activation control sample of asynchronous cells growing in exponential phase, treated or not with 10 mM H_2_O_2_. The histograms in red refer to samples treated with hydrogen peroxide, while gray histograms refer to parallel cultures grown in YPD. The percentage of cells with 2n and 4n DNA content is indicated for every time point.

In *C. albicans* both Hog1 and Mkc1 become activated upon oxidative stress (Alonso-Monge et al., 2003; Navarro-García et al., 2005), while Cek1 and Cek2 turn into a dephosphorylated state (Alonso-Monge et al., 2009; Correia et al., 2016). We therefore wondered whether activation of MAPKs by hydrogen peroxide could be dependent on the phase of the cell cycle. Exponentially growing cells of the clinical isolate SC5314 were therefore synchronized in G1, incubated at 37°C and samples collected each 30 min for oxidative challenge. In this assay 5 mM of hydrogen peroxide was used to assure full activation of the kinases (see Supplementary Figure 1), and cells were subjected to 5 min treatment before being processed. The pattern of MAPKs activation was analyzed by western blot with specific antibodies and the DNA content of the cells was quantified by flow cytometry after staining with propidium iodide (Figures 4A,B).

As shown in Figure 4A, both Mkc1 and Hog1 became activated upon 5 mM H_2_O_2_ addition at the analyzed cell cycle stage. However, Phospho-Hog1 and Phospho-Mkc1 levels were found to be higher at the initial cell cycle stages (at the G1/S/G2 transition), while the H_2_O_2_-induced dephosphorylation of Cek1was more pronounced at 95 and 125 min. Interestingly, in non-stressed cells at 35 min the basal levels of Phospho-Hog1 and Phospho-Mkc1 were higher, suggesting the importance of these kinases in the G1-S-G2 transition. At this time the addition of H_2_O_2_ restrains cells from budding (8% budded cells in treated cultures against 40% in non-treated cells) and cell cycle progression was also inhibited, albeit slightly, by the addition of the stressing agent (4n DNA content was observed in 17% of treated cells against 21% of non-treated cells) (Figure 4B). These results indicate that hydrogen peroxide triggers MAPKs activation independently of the cell cycle phase in which cells are during the challenge.

### Hydrogen peroxide stabilizes Sol1 in *hog1* mutants

In *S. cerevisiae*, Hog1 mediates cell cycle arrest by phosphorylating the cell cycle inhibitor Sic1 in a single residue at the carboxyl terminus, leading to the stabilization of the protein and to cell cycle arrest. Sic1 alleles without this motif do not arrest at G1, and become sensitive to osmotic stress (Escoté et al., 2004). In *C. albicans*, there are no evident Sic1 homologs based on primary sequence homology, but a related protein was identified by complementation of *dbf2* mutants (Atir-Lande et al., 2005). This protein, named Sol1 (Sic one like), participates in morphogenesis in *C. albicans*, being a substrate of the cell cycle ubiquitin ligase complex recognition component Cdc4. We therefore explored the possibility of Sol1 being a substrate of Hog1 to mediate cell cycle arrest in response to oxidative stress.

**Figure 5:**
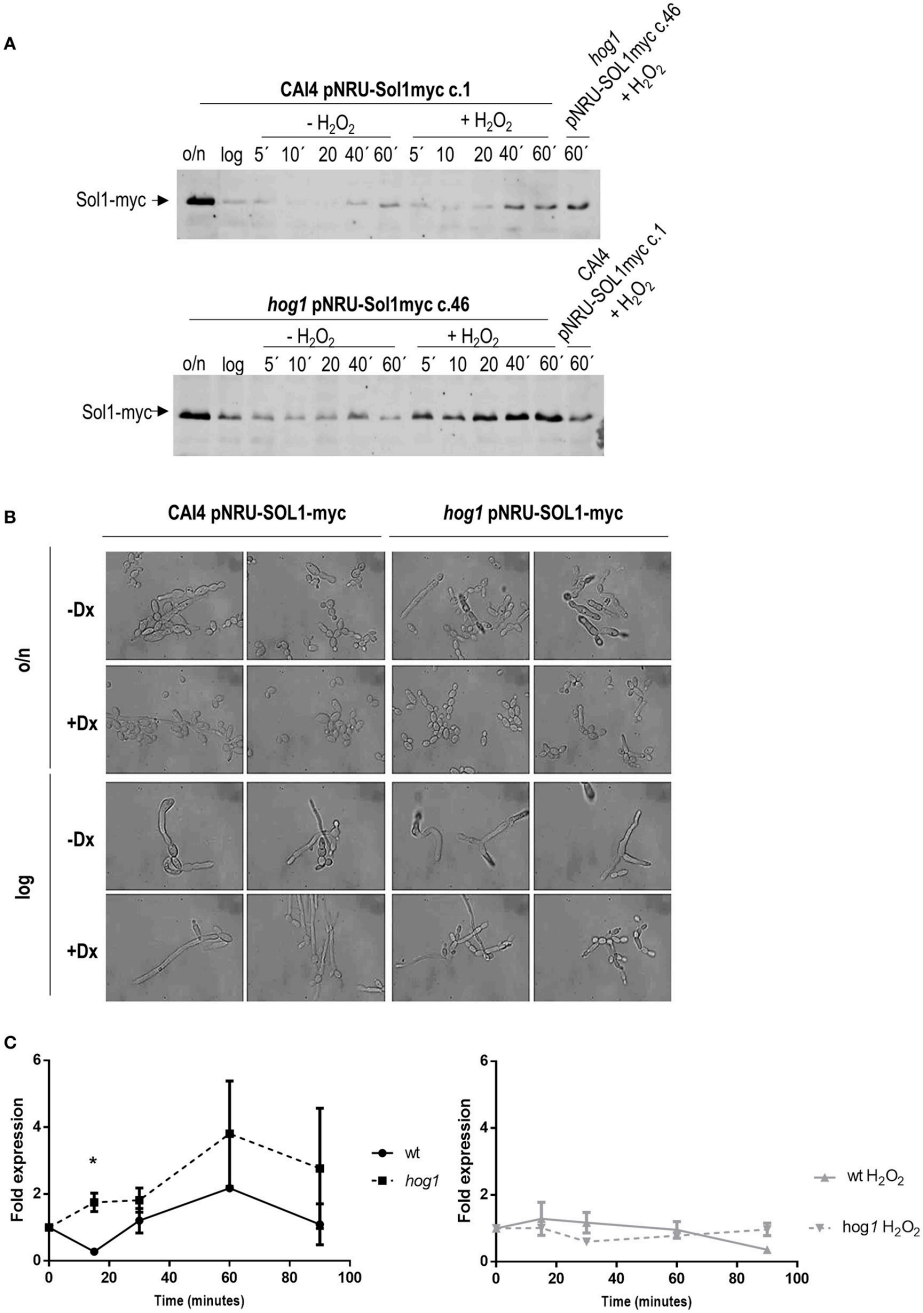
**Effect of hydrogen peroxide on Sol1 stability and expression**. Sol1 fused to myc was overexpressed in CAI4 and *hog1* mutant strains. **(A)** Samples from YPD cultures incubated at 37°C were taken in stationary phase (overnight indicated as o/n), diluted to O.D. 0.2 and grown for around 4 h until O.D. 1 was reached (logarithmic phase, log in the Figure). Cultures were split and treated or not with 5 mM H_2_O_2_. Samples were taken at the indicated time points and processed for Sol1-myc detection using anti-myc antibodies. Since the strains under analysis were analyzed in separated membranes, the sample from YPD plus stress at 60 min of the opposite strain was included in the right lane of the gel for a more precise comparison. **(B)** *C. albicans* strains overexpressing Sol1-myc were grown in the presence or not of doxycycline (Dx), which repress Sol1-myc expression. Morphology of the cells from stationary and exponentially growing cultures is shown. **(C)** Expression of Sol1 was quantified by Q-RT-PCR. CAF2 and *hog1* mutant cultures were elutriated and released in YPD with or without H_2_O_2_. Samples were taken at different time points and treated for Q-RT-PCR analysis. The *ACT1* mRNA was used as the internal control, and the fold of expression over each strain at time 0' (that is just after elutriation) is represented as a function of time. The graph on the left shows Sol1 expression in YPD cultures, while the graph on the right shows Sol1 expression levels in H_2_O_2_ treated cultures. ^*^ indicates significant differences between wild type and *hog1* mutant using unpaired *t*-test (*p*-value 0.0352). Graphs show the mean ± the standard error of the mean (SEM) of two independent experiments (2 replicates each).

We generated labeled Sol1 tagged with the myc epitope at the C-terminus and integrated this construct at the *ARD1 locus* of the CAI4 wild type strain (a *ura3* derivative strain of CAF2), as well as into the *hog1* mutant strain. *SOL1* expression was, in this case, controlled by the *OP4* promoter, repressed by doxycycline, therefore allowing the analysis of the stability of the protein independently of its native regulation. The stability of Sol1 was analyzed by western blot using antibodies against the myc epitope in the presence or absence of oxidative stress (Figure 5A). Although, we could not evidence a difference in electrophoretic mobility between peroxide treated and control cells in either the wild type or the *hog1* mutant strain, important differences were observed. Firstly, Sol1 accumulates in stationary (overnight) cells compared to exponentially growing cells. Secondly, addition of hydrogen peroxide stabilizes Sol1 compared with control cells without stress (Figure 5A). Thirdly, Hog1 mediates Sol1 degradation since the absence of Hog1 prevents Sol1 to degrade (Figure 5A lower panel) when cells resume growth. This effect was even stronger in the presence of oxidative stress, where in *hog1* mutant cells Sol1 accumulated to level observed in overnight cultures. These observations suggest a relationship between Hog1 and Sol1; however, we could not observe a physical interaction between Sol1-myc and Hog1 in a *C. albicans* adapted two hybrid systems (Supplementary Figure 2), suggesting that the interaction between these proteins may be indirect or so transient that is not detected by this methodology.

The ectopic expression of Sol1 has been reported to affect cell morphology (Atir-Lande et al., 2005). *C. albicans* strains carrying *SOL1*-myc under the control of a tetracycline repressed promoter were visualized under optical microscopy. The overexpression of *SOL1* led to morphological alterations, and both the wild type and the *hog1* mutant strains grew as aberrant filaments or pseudohyphae (Figure 5B). Addition of doxycycline repressed *SOL1* expression allowing cells to grow as yeast in overnight cultures, or form hyphae in exponential growing cultures. No differences in cell morphology were observed between wild type and the *hog1* mutant cells, indicating that the role of *SOL1* in morphogenesis is Hog1-independent.

The expression of *SOL1* was analyzed along the cell cycle. For this purpose, wild type and *hog1* mutant cultures were elutriated and G1 synchronized cells were released in YPD with or without H_2_O_2_ challenge at 37°C. Samples were taken at different time points and processed for quantitative PCR analysis. When wild type cells were released in YPD medium, *SOL1* mRNA dropped drastically at 15 min and then *SOL1* mRNA levels increased slowly to 2 fold increase relative to time point 0 at 60 min, and then decreased again at 90 min (Figure 5C, left graph). The mRNA expression pattern correlated with amount of the Sol1-myc fusion protein detected by western-blot (Figure 5A). This suggests that lowering *SOL1* mRNA levels could be important for cells to progress in the cell cycle. When the same culture was released into YPD medium in the presence of oxidative stress, *SOL1* mRNA increased slightly at 15 min and then maintained stable levels during 60 min although it eventually decreased at 90 min (Figure 5C, right graph). These results showed that oxidative stress led to a slight increase, followed by the maintenance of *SOL1* expression. Taken together transcription data and protein stabilization assays suggest that the presence of Sol1 prevents cell cycle progression upon hydrogen peroxide-induced stress. When *SOL1* expression level was quantified in the *hog1* mutant, no decrease was observed at 15 min post-release. The level of the *SOL1* transcript was higher in the *hog1* mutant compared to the wild type strain. Although these differences were not statistically significant except at 15 min (Figure 5C, left graph), they correlate with higher amount of the Sol1-myc fusion protein detected by western-blot (Figure 5A). In the presence of hydrogen peroxide, the amount of *SOL1* transcript remained unaltered in the *hog1* mutant during at least 90 min (Figure 5C, right graph). These data indicate that oxidative stress prevents oscillation in *SOL1* expression. Regulation of *SOL1* mRNA expression under oxidative challenge was independent of Hog1; nevertheless, Sol1 stabilization was Hog1-dependent as indicated by western analysis. Hog1 also may control *SOL1* mRNA expression during cell cycle progression, as in the absence of this MAPK, the *SOL1* mRNA transcript displayed higher levels in the *hog1* mutant compared to wild type cells (statistically significant at 15 min).

### The expression of G1 cyclins is dependent on Hog1

**Figure 6:**
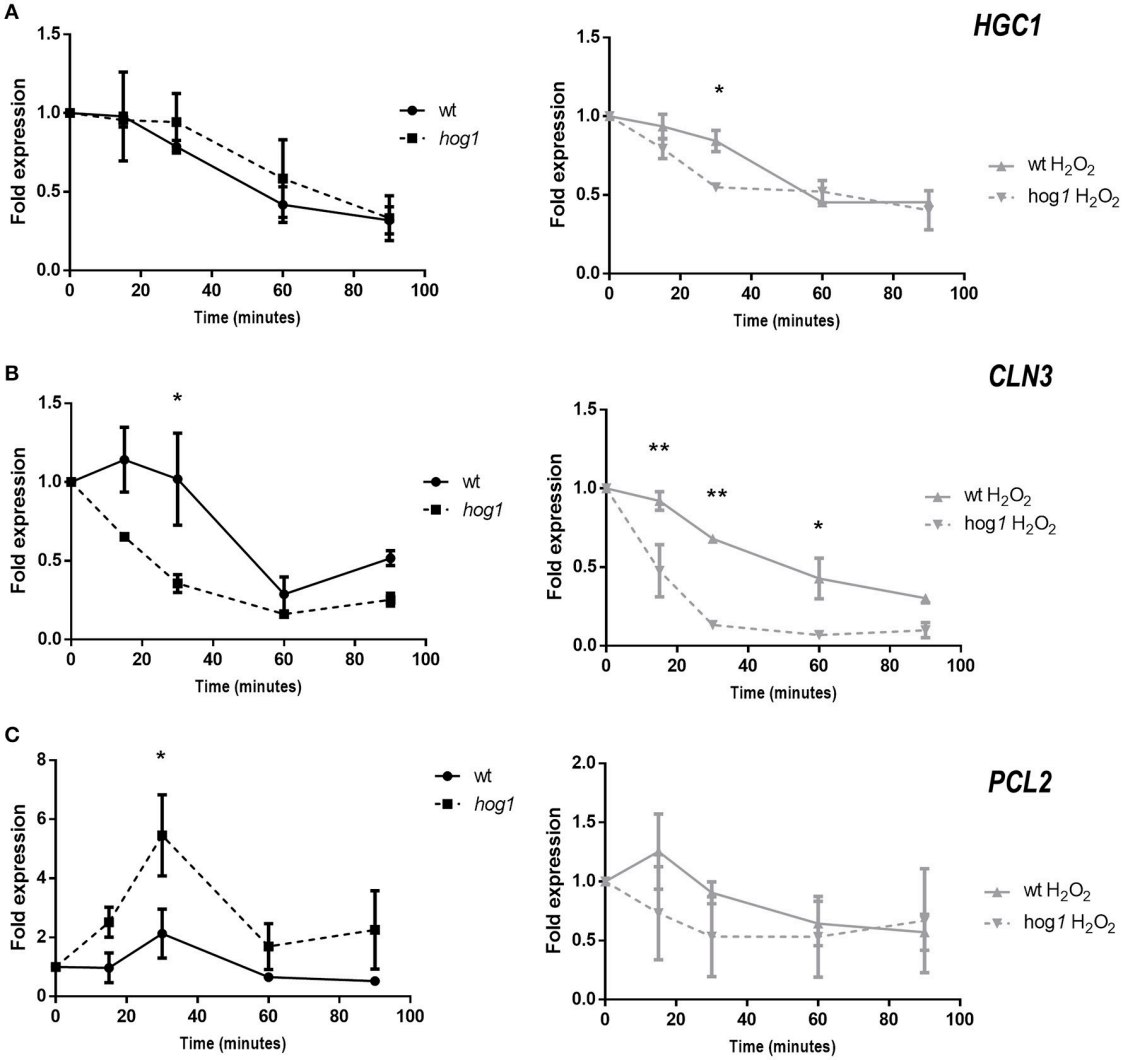
**Expression of cyclins in G1 synchronized cells released in the presence or absence of hydrogen peroxide**. CAF2 and *hog1* mutant cultures were elutriated and cells synchronized in G1 were released in YPD plus or minus H_2_O_2_(1 mM). Samples were taken at different time points and processed for Q-RT-PCR analysis. Expression of *HGC1* **(A)**, *CLN3* **(B)**, and *PCL2* **(C)** cyclins was quantified using *ACT1* mRNA as internal control, and the fold of expression over each strain at time 0 (just after elutriation) are represented along the time. Graphs on the left (black lines) show the cultures released in YPD, while graphs on the right (gray lines) show cultures released in the presence of oxidative stress. Significant differences were detected using 2-way ANOVA and Sidak's multiple comparisons test. Statistical differences are indicated (^*^p < 0.05, ^**^p < 0.001). Graphs show the mean ± the standard error of the mean (SEM) of two independent experiments (2 replicates each).

Since the expression of the cell cycle regulator Sol1 was altered in the *hog1* mutant, we next asked if the expression of cyclins was also Hog1-dependent. For this purpose, *C. albicans* cultures were elutriated and cells in G1 phase were released in YPD with or without H_2_O_2_ addition, at 37°C. Samples were taken at different time points and processed for quantitative PCR analysis. Expression of *HGC1, CLN3*, and *PCL2* was quantified. The expression levels of *HGC1*, a hypha-specific G1 cyclin, decreased over time in YPD cultures; no significant differences were detected between wild type and *hog1* mutant cells when each strain was compared with the basal level of gene expression in that strain (Figure 6A). When cells were released in the presence of H_2_O_2_, *HGC1* expression followed a similar kinetics in the wild type, suggesting that H_2_O_2_ does not influence *HGC1* expression in G1 cells. However, down regulation of *HGC1* was faster in the *hog1* mutant under oxidative stress. A significant difference was detected at 30 min after release, suggesting a role of Hog1 in *HGC1* transcription in the presence of stress (Figure 6A, right graph). Expression of the G1 cyclin *CLN3* was also quantified (Figure 6B). In the wild type, *CLN3* expression remained stable until 30 min and then decreased at 60 min. In the *hog1* mutant the downregulation of *CLN3* was faster than in the wild type, with significant differences observed at 30 min post- release. Significant differences between the wild type and the *hog1* mutant were observed for *CLN3* expression in the presence of oxidative stress. During oxidative stress, *CLN3* expression was repressed in the wild type as well as in the *hog1* mutant. However, this decrease was more drastic in the *hog1* mutant. Collectively, these data indicate that Hog1 plays a role in *CLN3* expression upon standard growth conditions, and in the presence of oxidative stress. Finally, *PCL2* expression was quantified (Figure 6C). A peak of expression of *PCL2* was detected at 30 min after release in the YPD medium. At this time point, the level of expression was significantly higher in the *hog1* mutant compared to the wild type strain, reaching a 5.46-fold higher transcript level relative to time point 0, compared with 2.13-fold up-regulation in the wild type. No significant differences were detected between strains when cultures released in the presence of H_2_O_2_ were compared. All these data indicate that Hog1 controls the expression of cell-cycle cyclins in the presence of oxidative stress, and/or under standard growth depending on the cyclin.

### Cell size is altered in *hog1* mutants

**Figure 7:**
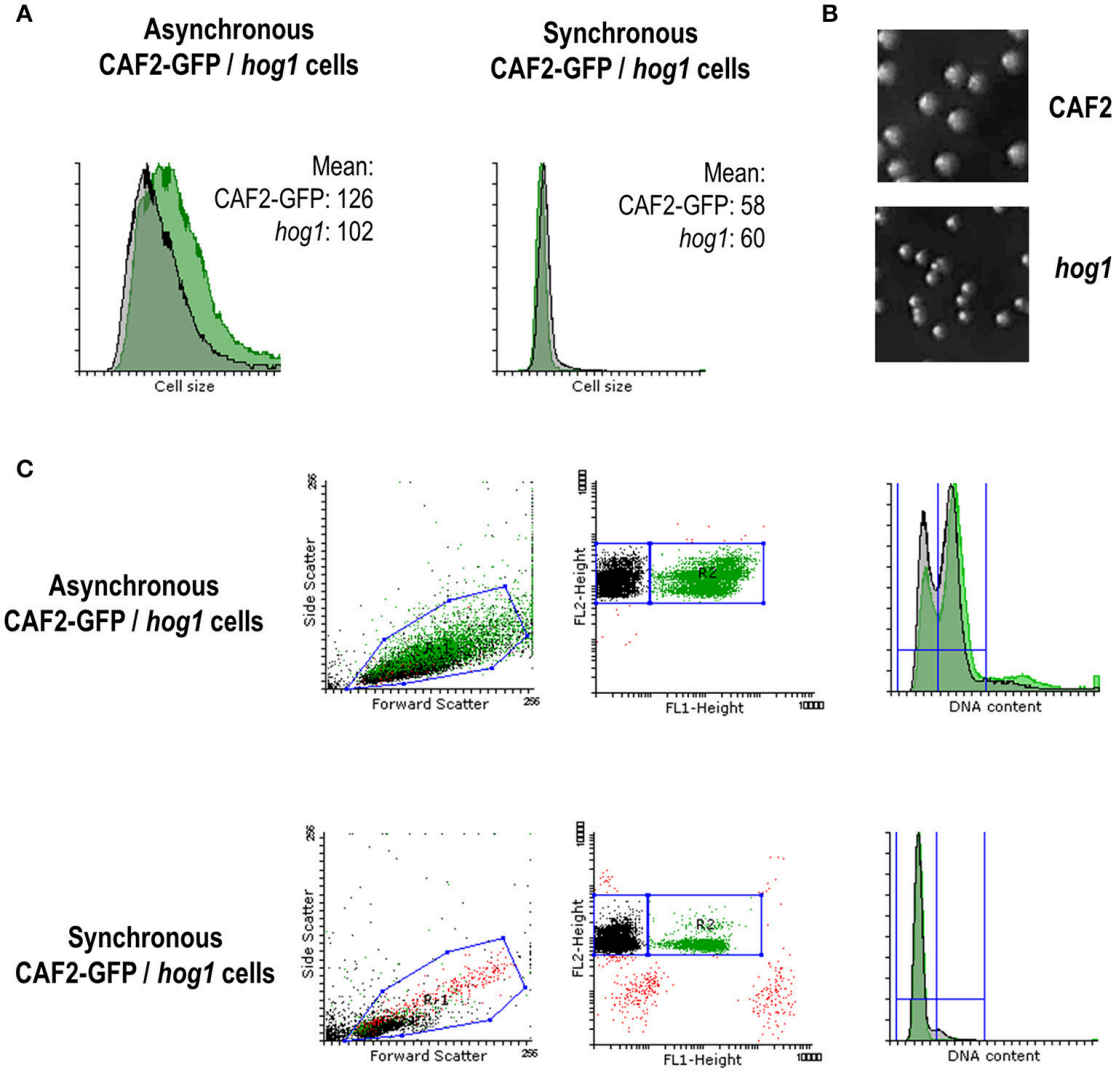
**Role of MAPKs in cell cycle progression and cell size. (A)** FACS histograms showing the mean cell size from asynchronous and synchronous (G1) mixed cultures of CAF2-GFP and *hog1* mutant. Cell size mean is specified in each case. **(B)** Pictures show colonies grown on YPD for 24 h at 37°C from CAF2 and *hog1* mutant strains. **(C)** Dot plots and histograms from mixed cultures before and after elutriation (referred as asynchronous and synchronous cells respectively). Forward and Side scatter dot plots show a unique population belonging to CAF2-GFP and *hog1* mutant mixed cultures. FL-1 (GFP) vs. FL-2 (PI) dot plots allows differentiation of two populations: green positive (CAF2) and green negative (*hog1* mutant cells). Both populations are PI positive. Histograms reflect their DNA content (FL-3) from asynchronous and synchronous cultures.

Given that Hog1 controls the expression of certain cyclins and cell cycle regulators (Figures 5C, 6) and that the cell cycle progresses faster in the *hog1* mutant compared to the wild type (Figure 3), we wondered if these features affected cell size. Cell size was quantified in either asynchronous cultures or in synchronous G1 cultures by flow cytometry (Figure 7A). Before elutriation CAF2-GFP cells appeared to be slightly bigger than *hog1* (cell size mean of 126.3 vs. 101.9 for the mutant strain). This was also observed on YPD solid plates, where *hog1* mutant colonies were persistently smaller that wild type's colonies (Figure 7B) and this was independent on the presence of oxidative stress.

Differences between the strains were not detected after elutriation since cells were selected by their size (Figure 7A right panel). Nevertheless, during the course of synchronization studies we observed a dissimilar proportion of elutriated cells in wild type/*hog1* mixed cultures. Dot plots (Figure 7C) show two easily distinguishable populations corresponding to CAF2 (GFP positive, 99% of the cells were labeled) and *hog1* (GFP negative, 0.06% false positive cells). Before elutriation (asynchronous cells) the mixed population was composed of 54% of GFP-positive cells (CAF2-GFP) and 44% of non-labeled cells (*hog1*), and both strains presented a mixed population with 2n and 4n DNA content (29% 2n, 59% 4n for CAF2-GFP and 34% 2n, 57% 4n for *hog1*). However, despite the fact that cell synchronization in G1 was effective (95% 2n, 3% 4n for CAF2-GFP and 93% 2n, 6% 4n for *hog1*) only 16% of the recovered population after elutriation corresponded to the GFP marked wild type strain CAF2 (contrasting with 80% *hog1*) (Figure 7C). This unbalanced mixed culture correlates with the smaller cell size displayed by the *hog1* mutant. Since *hog1* G1 cells are smaller than wild type G1 cells, *hog1* mutant cells were recovered in higher numbers from the mixed population. This reinforces the role of Hog1 in controlling cell cycle progression under basal conditions.

## Discussion

In eukaryotic organisms the cell cycle involves complex mechanisms required for cells to successfully divide. These processes need to be tightly coordinated, and the control of so-called checkpoints is crucial. Specific checkpoints enable progression or blockage of the cell cycle to avoid failure in the division process. Environmental stresses can cause macromolecular damage that alters DNA replication, abnormal chromosome segregation, cell death, and other problems. Given the role of MAPKs as a sensing system, it seems rather logical that these signaling proteins coordinate cell cycle progression in response to environmental cues. The budding yeast *S. cerevisiae* and the fission yeast *Schizosaccharomyces pombe* have been used as models to analyze cell cycle progression and understand control mechanisms. The role of MAPKs in cycle arrest and renewal of growth after stress adaptation has been demonstrated in these model fungi (reviewed by Correia et al., 2010). Nevertheless, this relationship has not been explored in the opportunistic pathogenic yeast *C. albicans*, probably due to the difficulty in obtaining synchronized populations which are required for cell cycle studies. Cell cycle arrest in *C. albicans* can frequently lead to morphological alterations: cells arrested at G1 phase tend to be more hyphal-like, whereas the arrest at S, G2 and M phases leads to pseudohyphal-like cells (Berman, 2006). We overcame this issue by maintaining a low cell density at every stage of the experiment, thus avoiding the potential release from the inhibition caused by quorum-sensing molecules present in the medium of stationary cells that could induce filamentation (Enjalbert and Whiteway, 2005). Germ tubes or hyphae were rarely, if ever, observed in our synchronization experiments.

As an opportunistic pathogen *C. albicans* has to face oxidative stress generated by the immune system. Although immune cells generate different reactive oxygen species, the response to hydrogen peroxide has been extensively studied. *C. albicans* responds to hydrogen peroxide by triggering Mkc1 and Hog1 phosphorylation and deactivating Cek1 (Navarro-García et al., 2001; Alonso-Monge et al., 2003, 2009; Correia et al., 2016). This behavior is not dependent on the cell cycle phase, as it is observed in different phases of the cell cycle, although the levels of Phospho-Mkc1 and Phospho-Hog1 seem higher when the oxidative challenge occurs at 5 and 35 min post release. This could reflect a higher need for cells to respond efficiently to oxidative stress at the beginning of cell division and when DNA is duplicating, as cells could be more vulnerable to oxidative damage at these stages of cell division. Interestingly, all analyzed MAPKs seem to display higher activation at the G1/S transition in basal conditions (without stress). Recently, an *MTLa* strain overexpressing the cyclin dependent kinase inhibitor *FAR1* was used to develop pheromone-induced cell cycle synchronization (Côte and Whiteway, 2008). This allowed the establishment of the global cell cycle expression profile of the mating competent (opaque) form of *C. albicans* (Côte et al., 2009). At the G1/S transition this strain displays a peak of *TRR1* expression, a thioredoxin reductase induced by peroxide, as well as several genes that are implicated in cell wall mannan biosynthesis (*ALG7, PMI1, GDA1*, and *MNN9*). Therefore, at this cell cycle stage, Hog1, Mkc1 and Cek1 could become activated to respond to potential ROS and to promote cell wall biogenesis.

In *S. cerevisiae*, both Hog1 and Slt2 have been shown to directly sense environmental stimuli to the checkpoints (reviewed by Correia et al., 2010). Hog1 can be activated by osmotic stress leading to cell cycle arrest at the G1/S and G2/M transition (Escoté et al., 2004; Clotet et al., 2006), while Slt2 is responsible for arresting the cell cycle at G2 upon alteration of cell integrity (Harrison et al., 2001). Oxidative stress can also result in cell cycle arrest in *S. cerevisiae*: menadione arrests cells at the G1 phase, whereas hydrogen peroxide was suggested to cause a G2 arrest by an alternate mechanism from that affected by menadione (Flattery-O'Brien and Dawes, 1998) and to cause a delay in the S phase (Leroy et al., 2001). Although the phosphorylation of Hog1 in response to hydrogen peroxide has been reported in *S. cerevisiae*, no connection with the Mec1 DNA damage checkpoint pathway was detected (Haghnazari and Heyer, 2004). In contrast to *S. cerevisiae*, cell cycle progression in *C. albicans* is arrested at G1 by hydrogen peroxide. This arrest is not entirely dependent on Hog1, as *hog1* mutants are still able to stop in the cell cycle. However, the absence of *HOG1* leads to a delay in cell cycle progression after oxidative challenge. The use of GFP to label *C. albicans* cells allowed us to design experiments where both wild type and mutant strains are processed at the same time. This novel strategy strengthens our observations from single strain cultures, demonstrating that Hog1 plays a relevant role in the resumption of cell cycle after oxidative stress. Moreover, in *C. albicans* the importance of Hog1 in cell cycle progression does not seem to be restricted to oxidative sensing, as in the absence of stress *hog1* mutants seem to progress more rapidly in the cell cycle than the wild type. This observation could explain why *hog1* cells and *hog1* forming colonies in YPD medium seem smaller when compared to other strains, and why, when elutriating an exact mixture of CAF2 and *hog1* cells, the number of *hog1* cells in synchronized G1 phase cells fluid is always higher. This faster cell cycle progression was exclusive of mutants in the HOG pathway (observed in both *hog1* and *pbs2* defective mutant - data not shown), since it was not detected in *mkc1* or *cek1* mutants. Cell size is an important feature for cell physiology. In *S. cerevisiae*, cell size depends on nutrient availability and is controlled, primarily, at *START* where the amount of the G1 cyclin Cln3 is crucial for cell size and cell cycle progression (Turner et al., 2012). Cln3 forms part of the CDK1 complex which activates the G1-S transcription factor SBF leading to the entry into S-phase. Here, we report that the expression of *CLN3* in the *C. albicans* wild type strain remains constant during 30 min after cells being released at G1, and drops at 60 min which concurs with G2/M phase. Nevertheless, in the absence of Hog1, *CLN3* downregulation occurs earlier, this decrease in the *CLN3* expression could lead to a sooner entry into S phase and to a faster cell cycle observed in the absence of stress in the *hog1* mutant. *CLN3* is the only essential G1 cyclin in *C. albicans* being also important in cell size control at G1 and in the timing of the transition to hyphal growth. Depletion of Cln3 arrests cell in G1 which cause cells enlarged in size unable to bud that form filamentous spontaneously to resume the cell cycle (Bachewich and Whiteway, 2005; Chapa y Lazo et al., 2005). In *C. albicans* the expression of *CLN3* is reduced in the presence of farnesol (Enjalbert and Whiteway, 2005) a *quorum sensing* molecule that inhibits hyphal growth and triggers Mkc1, Hog1, and Cek1 activation (Smith et al., 2004; Román et al., 2009). Thus, environmental signal connects signal transduction pathways with cell cycle regulation.

*CLN3* expression displayed a similar pattern in the presence of hydrogen peroxide to control cultures although no progression in the cell cycle occurred. Whether *CLN3* expression controls cell cycle progression or mediates an arrest remains undetermined, as other cyclins and cyclin dependent kinases (CDK) are involved in cell cycle progression. In fact, *PCL2* expression decreased in Cln3-depleted cells that are forming hyphal-like extensions (Bachewich and Whiteway, 2005), and the presence of farnesol prevents repression of *PCL2* while it prevents induction of *HGC1* (Enjalbert and Whiteway, 2005). Also, and contrary to *HGC1, PCL2* is mainly expressed in yeast cells rather than hyphae (Enjalbert and Whiteway, 2005; Ihmels et al., 2005), which suggests a complementary role for these cyclins in yeast and hyphal cells, given that Hgc1 associates with Cdc28 CDK (Zheng et al., 2004) and Pcl2 is predicted to associate with the Pho85 CDK. Previously, a Pcl2 expression peak at G1/S phase was reported (Côte et al., 2009) and we detected this peak after 30 min following release of G1 cells into YPD. Our data indicate that Hog1 represses *PCL2* expression since elevated *PCL2* transcript levels were detected in the absence of *HOG1*. No significant differences were observed between wild type and *hog1* mutant strains when G1 phase cells were released in the presence of H_2_O_2_ during the period of time analyzed.

The expression pattern of *HGC1* was similar in both strains in the absence of stress. *HGC1* encodes a protein involved in regulating mycelial growth (Zheng et al., 2004; Fan et al., 2013), *HGC1* is expressed in hyphae and not in yeast and is negatively regulated by the transcriptional repressors Nrg1 and Tup1 (García-Sanchez et al., 2005). Although required for true hyphal formation, *hgc1* mutants are able to develop pseudohyphae and express hypha-associated genes and adhesins (Zheng et al., 2004). The fact that the cells kept unicellular yeast morphology could explain why the expression of *HGC1* was repressed in our experiments. On the other hand, hydrogen peroxide in either sub-toxic (1 mM) or toxic levels (10 mM) was seen to induce pseudohyphal growth in *C. albicans* by different mechanisms (Nasution et al., 2008; Srinivasa et al., 2012). Additionally, Hog1 acts as a repressor of filamentation (Alonso-Monge et al., 1999, 2003). Under our experimental conditions neither Hog1 nor hydrogen peroxide induced *HGC1* expression. This fact is in agreement to the unicellular morphology observed.

Sic1 is a cyclin-dependent kinase inhibitor, which in *S. cerevisiae* acts by inhibiting the Cdc28-Clb kinase complexes that controls the G1/S phase transition. Under osmotic shock, Hog1 controls cell cycle progression in *S. cerevisiae* directly phosphorylating Sic1 to avoid degradation (Escoté et al., 2004). Sol1 was identified as functional homolog of *S. cerevisiae* Sic1 in *C. albicans*. In *C. albicans* Sol1 may play a similar role to Sic1, as it has been reported that Sol1 inhibits CaClb4 and CaClb2 and that CaSol1 at high concentration is itself a substrate of both CaClb2 and CaClb4 (Ofir and Kornitzer, 2010). We show that Sol1 accumulates in the presence of oxidative challenge. Moreover, Sol1 is also regulated at the transcriptional level. Expression of Sol1 decreases at 15 min, and increases later showing a peak at 60 min. This pattern correlates with the cyclic expression reported by Côte et al. (2009). Oxidative stress prevents the variation in expression concurring with cell cycle arrest. These data indicate that oxidative stress modulates both the expression of the gene and the stabilization of Sol1 protein probably to mediate cell cycle arrest. Contrary to what occurs in *S. cerevisiae*, the lack of Hog1 leads to stabilization of the Sol1 protein as well as enhanced transcription demonstrating that Hog1 mediates cell cycle progression both in the presence and absence of stress. Ectopic expression of *SOL1* led to elongated buds. Moreover, *SIC1* overexpression in *S. cerevisiae* enhances invasive growth on nitrogen starvation medium (Atir-Lande et al., 2005; Shively et al., 2013). Thus, Sol1 and its paralog Sic1 play a role in morphogenesis; this role is Hog1 independent since the absence of Hog1 does not prevent Sol1-dependent pseudohyphal-induced growth.

In conclusion, our results demonstrate a role for the MAP kinase Hog1 in cell cycle progression in *C. albicans*, therefore highlighting its potential usefulness as a target in controlling the proliferation of this microorganism.

## Author contributions

IC performed experimental work, discussion and writing. JP, work supervision, discussion and writing. RA, work supervision, discussion and writing.

### Conflict of interest statement

The authors declare that the research was conducted in the absence of any commercial or financial relationships that could be construed as a potential conflict of interest.

## Supplementary material

Supplementary Figure 1 **Threshold of activation for MAPKs upon hydrogen peroxide exposure**. Exponentially growing cells from the SC5314 wild type strain were exposed to the indicated H_2_O_2_ concentrations and processed for western-blot. Phosphorylated forms of Hog1, Mkc1, and Cek1 were detected using specific antibodies and indicated as P-Hog1, P-Mkc1, and P-Cek1. The total amount of Hog1 was detected using the anti-Hog1 antibody and used as the loading control.

Supplementary Figure 2 **Sol1-myc and Hog1 do not interact in a two-hybrid system**. Hog1 and Sol1 were fused to either LexA (DNA-binding domain) or VP16 (activation domain) for interaction studies through a two-hybrid system. Strains with only one of the fused constructs were used as negative controls and a strain with both LexA-Cek2 and VP16-Cek1 constructs was used as positive control for interaction (Stynen et al., 2010). Cells from overnight cultures were adjusted at OD = 0.8 and tenfold serial dilutions were spotted on histidine/methionine depleted SD or complete SD medium to test for their ability to stimulate *HIS1* expression. Two representative clones from each strain are shown. Plates were incubated for up to 5 days at 37°C (shown are days 1 and 4).
