# Corrigendum: The Hog1 MAP Kinase Promotes the Recovery from Cell Cycle Arrest Induced by Hydrogen Peroxide in *Candida albicans*

**DOI:** 10.3389/fmicb.2017.00555

**Published:** 2017-04-13

**Authors:** Inês Correia, Rebeca Alonso-Monge, Jesús Pla

**Affiliations:** Departamento de Microbiología II, Facultad de Farmacia, Universidad Complutense de MadridMadrid, Spain

**Keywords:** *Candida albicans*, cell cycle, oxidative stress, signaling, MAPK

In the original article, there was a mistake in Figure 2C as published. Histograms in the lower row did not belong to the experiment described in the manuscript. The corrected Figure 2 appears below. The authors apologize for this error and state that this does not change the scientific conclusions of the article in any way.

**Figure 2:**
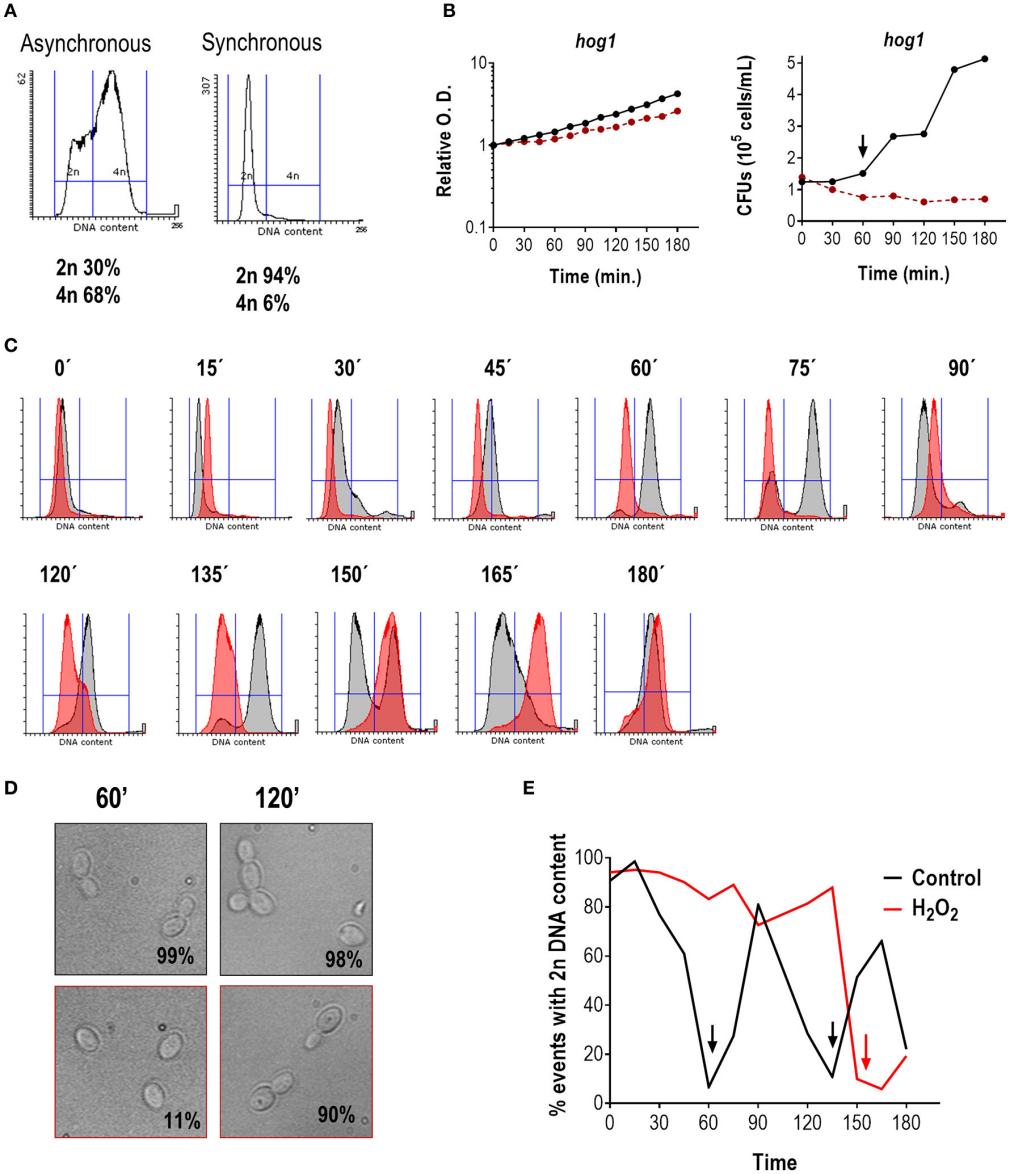
**Effect of H_2_O_2_ on cell cycle progression in the hog1 mutant. (A)** Flow cytometry analysis of hog1 mutant cells before and after elutriation. The percentage of cells with 2n and 4n DNA content are indicated. **(B)** After elutriation, G1 synchronized cells were released in YPD at 37°C with or without 1 mM H_2_O_2_. The growth was followed over time and depicted as relative O.D. (left panel) or CFUs vs. time (right panel). The black line represents cultures without stress, while the red line represents cultures in the presence of H_2_O_2_. The arrow marks cytokinesis. **(C)** Histograms of cultures released in the presence (red) or absence (gray) of oxidative stress at the indicated time. **(D)** Representative pictures of the culture without stress (upper row, black frame) or with an oxidative agent (1 mM H_2_O_2_, lower row, red frame). The percentage of budding is indicated for each condition. **(E)** The percentage of cells with 2n DNA content is plotted vs. time for cultures released with and without stress. Arrows indicate 4n DNA content.

## Conflict of interest statement

The authors declare that the research was conducted in the absence of any commercial or financial relationships that could be construed as a potential conflict of interest.

