# Supplementary figures and images for: The Hog1 MAP Kinase Promotes the Recovery from Cell Cycle Arrest Induced by Hydrogen Peroxide in Candida albicans
Source: Front Microbiol. 2017 Jan 6;7:2133. doi: 10.3389/fmicb.2016.02133 (PMC5216027; doi:10.3389/fmicb.2016.02133)

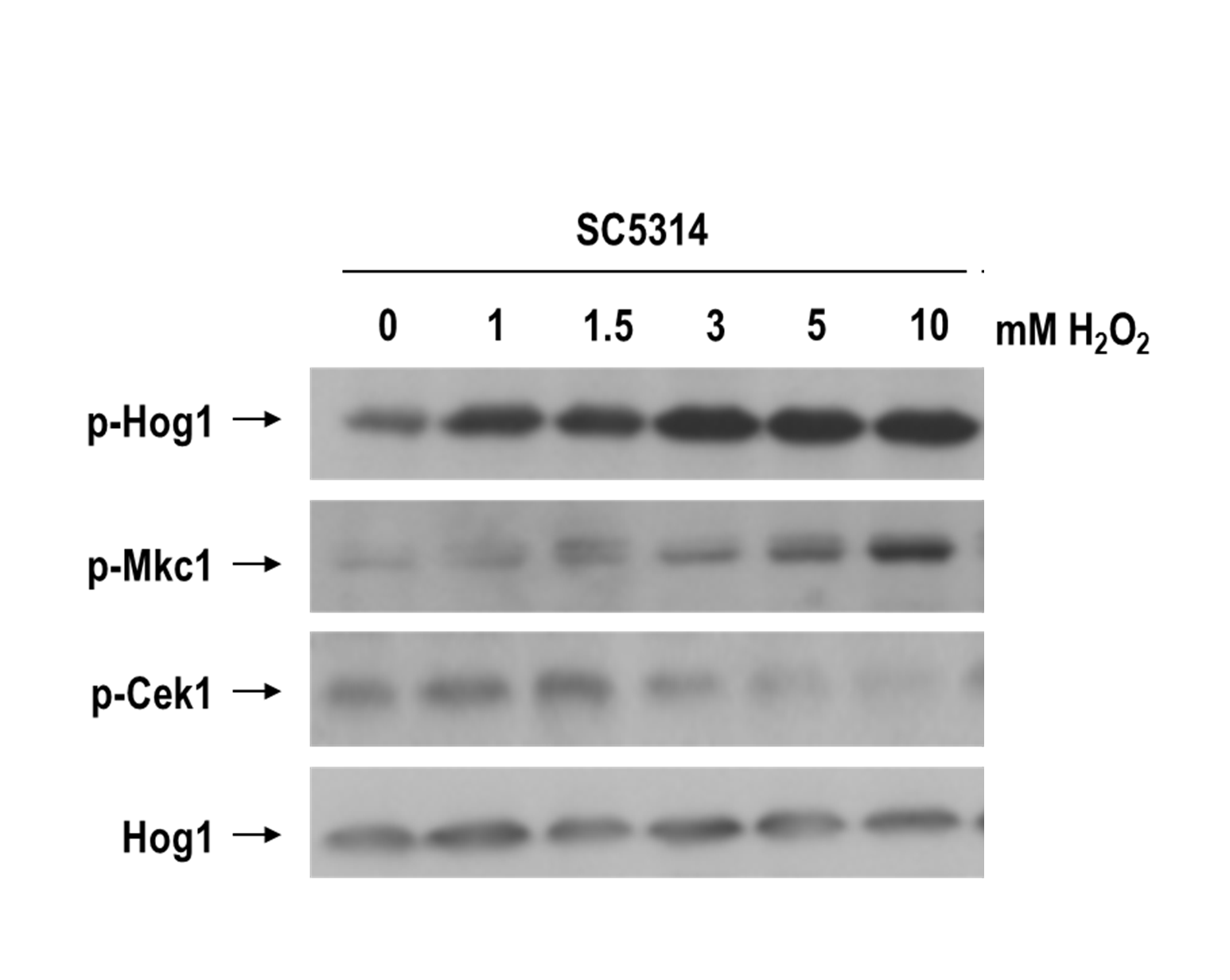

Supplement: Supplementary Figure 1 — Threshold of activation for MAPKs upon hydrogen peroxide exposure. Exponentially growing cells from the SC5314 wild type strain were exposed to the indicated H2O2 concentrations and processed for western-blot. Phosphorylated forms of Hog1, Mkc1, and Cek1 were detected using specific antibodies and indicated as P-Hog1, P-Mkc1, and P-Cek1. The total amount of Hog1 was detected using the anti-Hog1 antibody and used as the loading control. [file Image1.TIF]

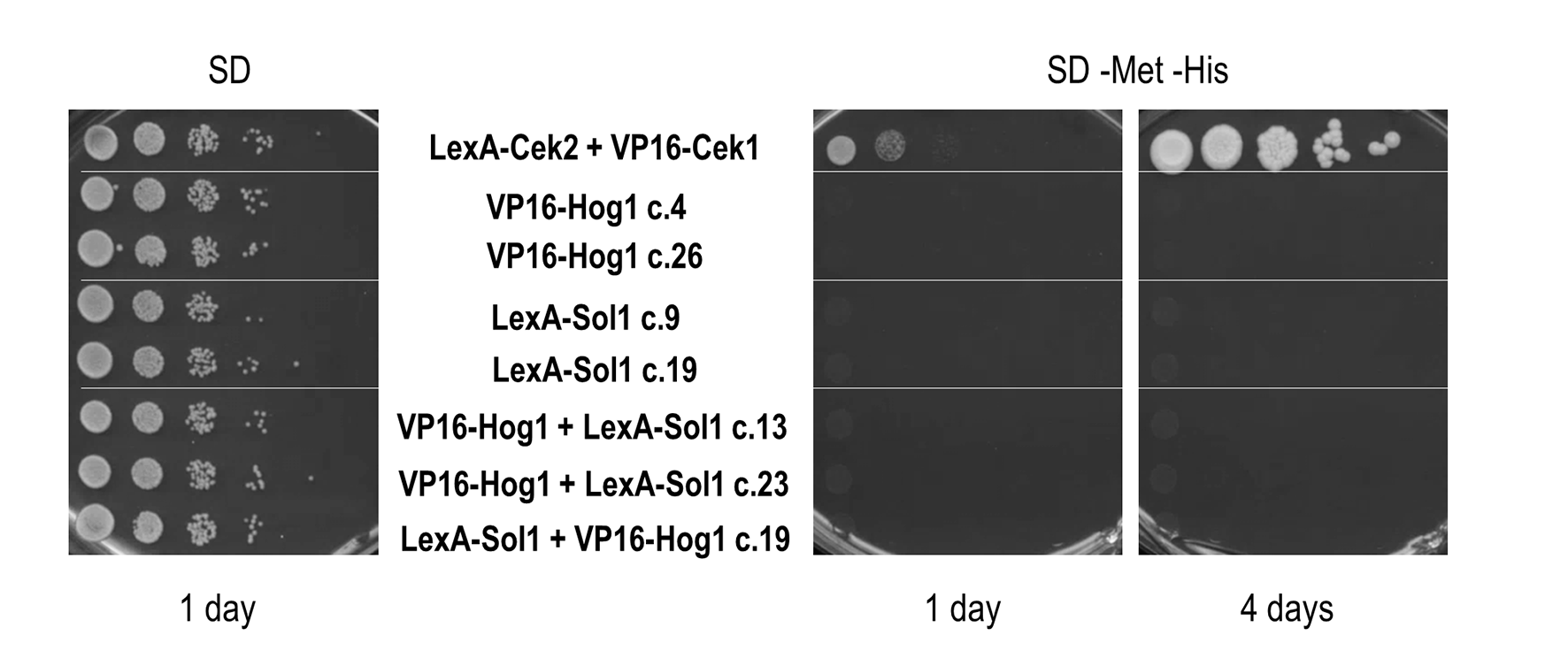

Supplement: Supplementary Figure 2 — Sol1-myc and Hog1 do not interact in a two-hybrid system. Hog1 and Sol1 were fused to either LexA (DNA-binding domain) or VP16 (activation domain) for interaction studies through a two-hybrid system. Strains with only one of the fused constructs were used as negative controls and a strain with both LexA-Cek2 and VP16-Cek1 constructs was used as positive control for interaction (Stynen et al., 2010). Cells from overnight cultures were adjusted at OD = 0.8 and tenfold serial dilutions were spotted on histidine/methionine depleted SD or complete SD medium to test for their ability to stimulate HIS1 expression. Two representative clones from each strain are shown. Plates were incubated for up to 5 days at 37°C (shown are days 1 and 4). [file Image2.TIF]
